# Glial Tissue Mechanics and Mechanosensing by Glial Cells

**DOI:** 10.3389/fncel.2018.00025

**Published:** 2018-02-21

**Authors:** Katarzyna Pogoda, Paul A. Janmey

**Affiliations:** ^1^Department of Physiology, University of Pennsylvania, Philadelphia, PA, United States; ^2^Institute of Nuclear Physics, Polish Academy of Sciences, Krakow, Poland

**Keywords:** brain tissue rheology, mechanical properties of brain tumors, brain-mimicking ECMs, mechanosensing, normal and transformed glial cells

## Abstract

Understanding the mechanical behavior of human brain is critical to interpret the role of physical stimuli in both normal and pathological processes that occur in CNS tissue, such as development, inflammation, neurodegeneration, aging, and most common brain tumors. Despite clear evidence that mechanical cues influence both normal and transformed brain tissue activity as well as normal and transformed brain cell behavior, little is known about the links between mechanical signals and their biochemical and medical consequences. A multi-level approach from whole organ rheology to single cell mechanics is needed to understand the physical aspects of human brain function and its pathologies. This review summarizes the latest achievements in the field.

## Introduction

The mammalian central nervous system consists of two major structures: the brain and the spinal cord, that are connected through brain stem. The brain is among the softest tissues of our body and is encapsulated by the skull, which provides mechanical protection for the brain. Mechanical properties of brain tissue play important roles in its development, physiology, and pathology and contribute significantly to neuromechanical signaling by mediating the effects of physical stimuli on brain function. During the past years, efforts have been made to understand the mechanical properties of the brain as a material by characterizing its storage and loss moduli, and how these moduli change as a function of strain rate, shear, compression, or tension. Owing to the fact that all tissues consist of cells surrounded by extracellular matrix, it is important to understand the mechanical characteristics of the brain tissues also at the single cell level and elucidate the mechanoresponse of these cells when in contact with ECM-mimetic platforms.

## Mechanical properties of the brain

The clear response of both normal and malignant cells of the central nervous system to modifications of their substrate suggests that changes in mechanical properties of brain that occur as a result of injury or disease might be an important factor in progression of the disease process, and are not simply a consequence of the pathological tissue structure. In addition, change of brain stiffness in these pathologic states can be a useful diagnostic marker of pathology, and if it were possible to measure local mechanics noninvasively, the need for risky invasive procedures could be reduced. Considerations such as these have led to many recent studies that measure the mechanical properties of brain using a large variety of techniques to impose different magnitudes and timescales of deformation, and which can lead to results that are likely complementary to each other, but that are currently difficult to relate.

**Figure 1:**
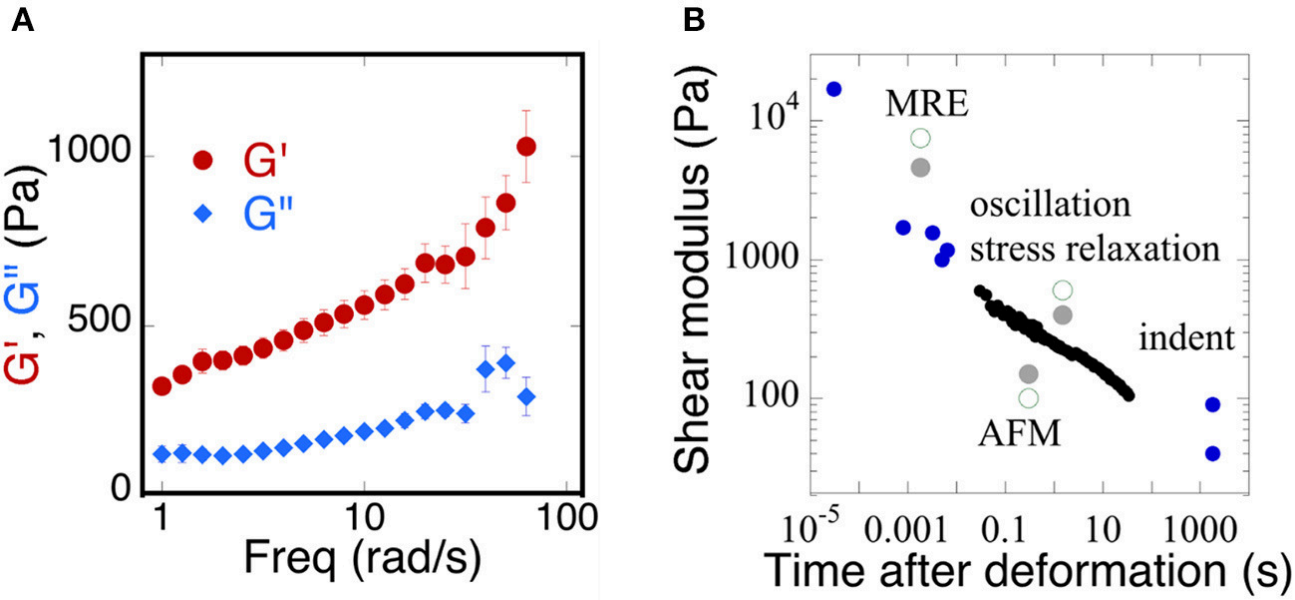
Time dependence of the viscoelastic properties of brain. **(A)** Replotted with permission from Pogoda et al. (2014) **(B)** adapted with permission from Franze et al. (2013) (copyright 2013, Annual Reviews), black circles represent shear moduli measured by stress relaxation, blue circles represent a variety of MRE, oscillation, indentation, and AFM methods, gray solid and green open circles represent a direct comparison of the measurements taken from gray or white matter of the brain, respectively.

Early rheological studies of brain mechanics used macroscopic samples of tissue derived from different areas of the brain and deformed them in shear using conventional or modified rheological instrumentation to report time-dependent shear moduli in which the shear stress decays with time after imposition of a constant deformation, or else using oscillatory measurements over a range of frequencies, usually limited at the high-end to a few tens of Hertz. More recently ultrasound elastography and especially magnetic resonance elastography (MRE) have been widely applied to brain (Guo et al., 2013; Braun et al., 2014) in order to determine whether local changes in mechanical properties might arise during development of cancer (Streitberger et al., 2014; Yang et al., 2014; Reiss-Zimmermann et al., 2015; Chauvet et al., 2016; Pepin et al., 2017), Alzheimer's disease (Hiscox et al., 2016; Murphy et al., 2016; Munder et al., 2017), other neurodegenerative diseases (Weickenmeier et al., 2017), inflammation (Jamin et al., 2015; Fehlner et al., 2016), or traumatic injury (Schmidt et al., 2016; Feng et al., 2017; Moeendarbary et al., 2017). These studies apply either compressive pressure waves or shear waves that displace the brain tissue at very small strains but much higher frequencies than are usually probed by conventional rheometry. As a result, the magnitudes of shear storage and shear loss moduli that are reported in different studies to quantify the material properties of brain are strongly dependent on timescale, but their precise functional dependence on frequency is often not known and can vary strongly from one condition to another. An example of the range of values reported is summarized in Figure 1 which shows (Figure 1A) the dependence of the shear modulus of a slice through a whole mouse brain measured in oscillatory deformation (Pogoda et al., 2014) and (Figure 1B) a summary of many values taken from the literature from a variety of measurement methods that operate at different time scales (Franze et al., 2013).

The magnitude of shear elastic modulus varies by more than two orders of magnitude when samples are deformed at fast rates that are relevant to traumatic injury or slow rates that are relevant to the slow movements of neurons and glial cells that mechanosense during development and repair. Also striking is the fact that the shear modulus does not appear to reach a steady value at very long time scales, suggesting that, as concluded in an earlier study (Bilston et al., 1997) brain responds to mechanical stress as a viscoelastic fluid rather than a solid like other tissues that are not held within a rigid boundary like the skull. The dissipative fluid-like rheology of normal brain suggests that changes in viscous dissipation might be as important as changes in elastic moduli and therefore might be applied as potentially useful diagnostic data.

### Brain softening in glial scar after trauma

**Figure 2:**
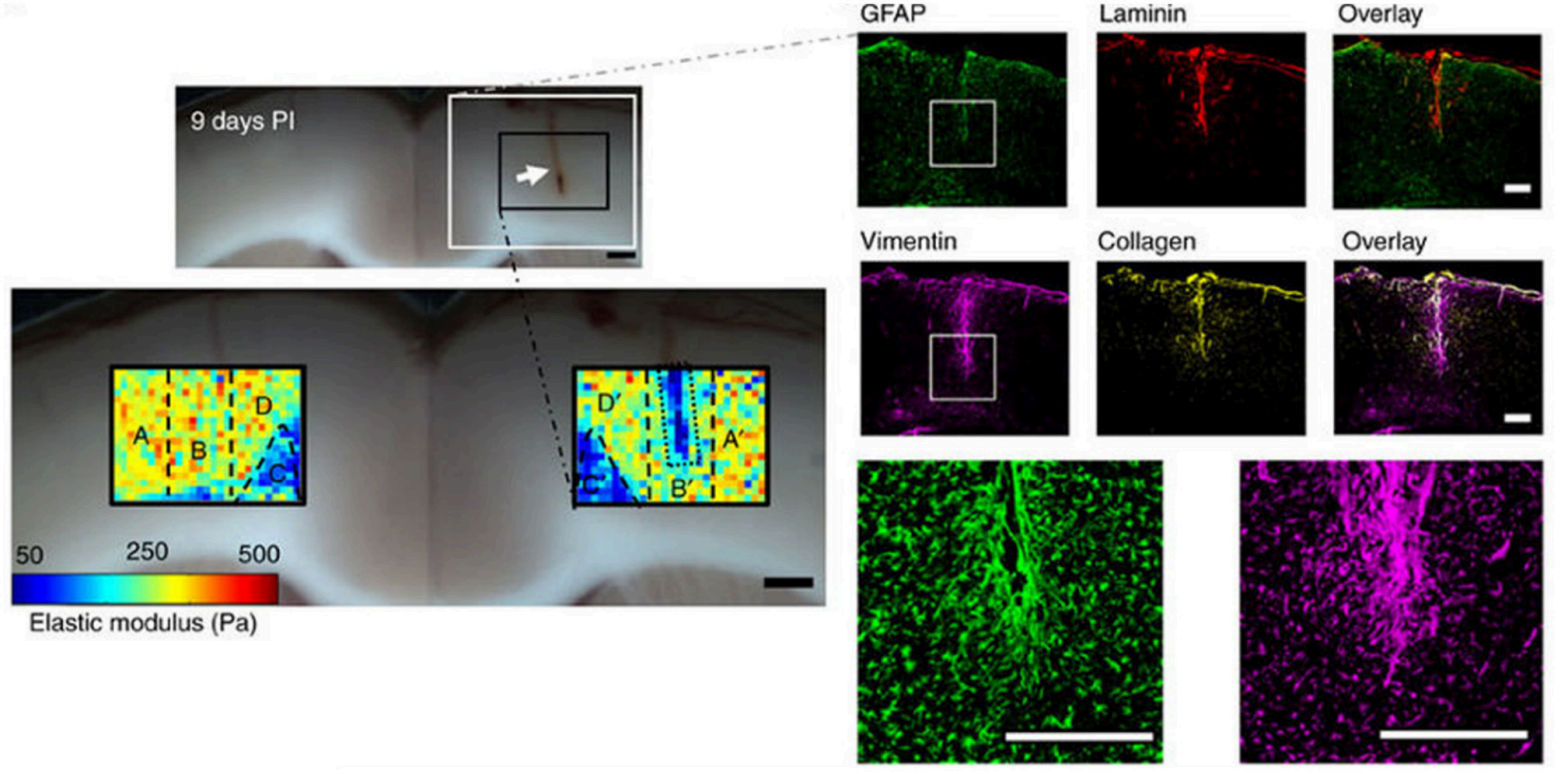
Softening of glial scars. A 2 mm stab injury (white arrow in the top bright field image) was induced in the cortex of the rat brain. The color maps below show the spatial distribution of elastic moduli in the injured and contralateral hemispheres 9 days after the injury. At the same time, immunostaining showed upregulation of vimentin and collagen, which are markers of the glial scar formation. Adapted with permission from Moeendarbary et al. (2017).

A striking and unexpected change in brain stiffness occurs subsequent to traumatic injury, such as a stab wound, that produces a glial scar (Moeendarbary et al., 2017). Even the term glial scar implies a stiffening of the injured region, but until very recently the mechanical properties of the glial scar had not been measured. A recent study using atomic force microscopy indentation to determine the elastic modulus of the injured site showed that 9 days after injury, a time when molecular markers of glial scarring were strongly upregulated, the glial scar was surprisingly much softer rather than stiffer than the area around it (Figure 2). This finding is especially surprising because the glial scar contains increased concentrations of fibrillar collagen, an extracellular matrix component not usually found in normal brain, which normally has an extracellular matrix composed of much softer polymers such as glycosaminoglycans (GAGs). The finding that glial scars are softer rather than stiffer than normal CNS has important implications for possible therapeutic approaches, since previous work hypothesized that the stiffened region inhibited the entry of protrusions from surviving neurons, since neurite protrusion and branching occurs more on soft substrates *in vitro* (Flanagan et al., 2002). It is likely that some other compound of the newly formed extracellular matrix or cellular environment elicits the signals that make axonal infiltration into the injury site difficult, and it is not simply the stiffening of the lesion site that acts as a barrier.

### Mechanical properties of brain tumors

**Figure 3:**
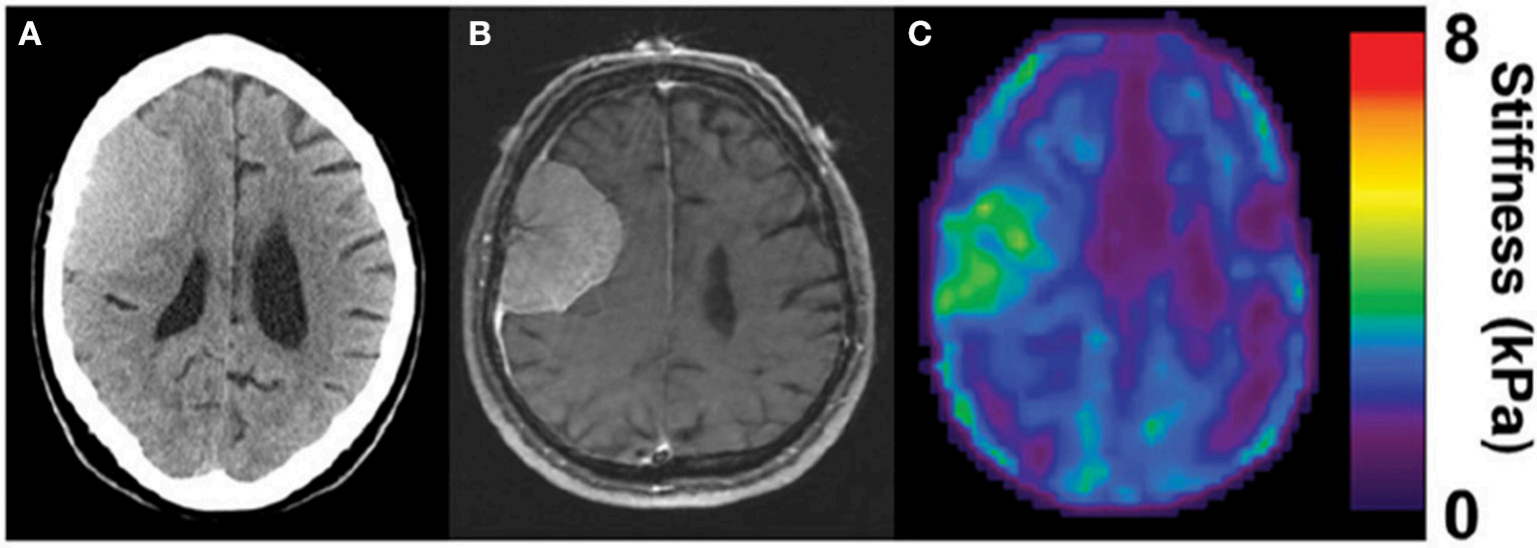
Stiffening of meningioma as measured by MRE. Adapted from Hughes et al. (2015) by permission of Oxford University Press. **(A)** CT image of the head, **(B)** corresponding MRI, and **(C)** corresponding MRE image showing homogenous tumor with the stiffness greater than surrounding healthy tissue (in green).

Several types of tumors, notably those of breast and colorectal cancers, are stiffer than the surrounding area (Butcher et al., 2009), and this abnormal stiffening is commonly used for diagnosis by palpation and other methods. In other types of cancer such as liver, a stiffened liver due to pre-existing fibrosis is a very strong risk factor for eventual development of hepatocellular carcinoma, suggesting that early detection of stiffening would be valuable for screening or monitoring disease progression. Similar considerations have been led to use of mechanical measurements, often by MRE to visualize noninvasively gliomas and other brain tumors, and thereby aid treatment and surgical resection. The hypothesis supporting these endeavors is that brain tumors also have mechanical properties distinct from those of the surrounding tissue. In some cases, strong evidence has been provided that this potential can be realized in some settings. For example, Figure 3 shows a magnetic resonance elastogram from a patient with a meningioma, in which the elastography clearly delineates a region coincident with the tumor that shows that the diseased region is significantly stiffer than the surrounding brain (Hughes et al., 2015). Not all types of tumors even of the same class of cancer appear to follow this pattern, and it remains to be seen how universal a change in stiffness is at sites of tumor growth, and whether noninvasive elastography can be as useful in brain tumors is in other settings.

**Figure 4:**
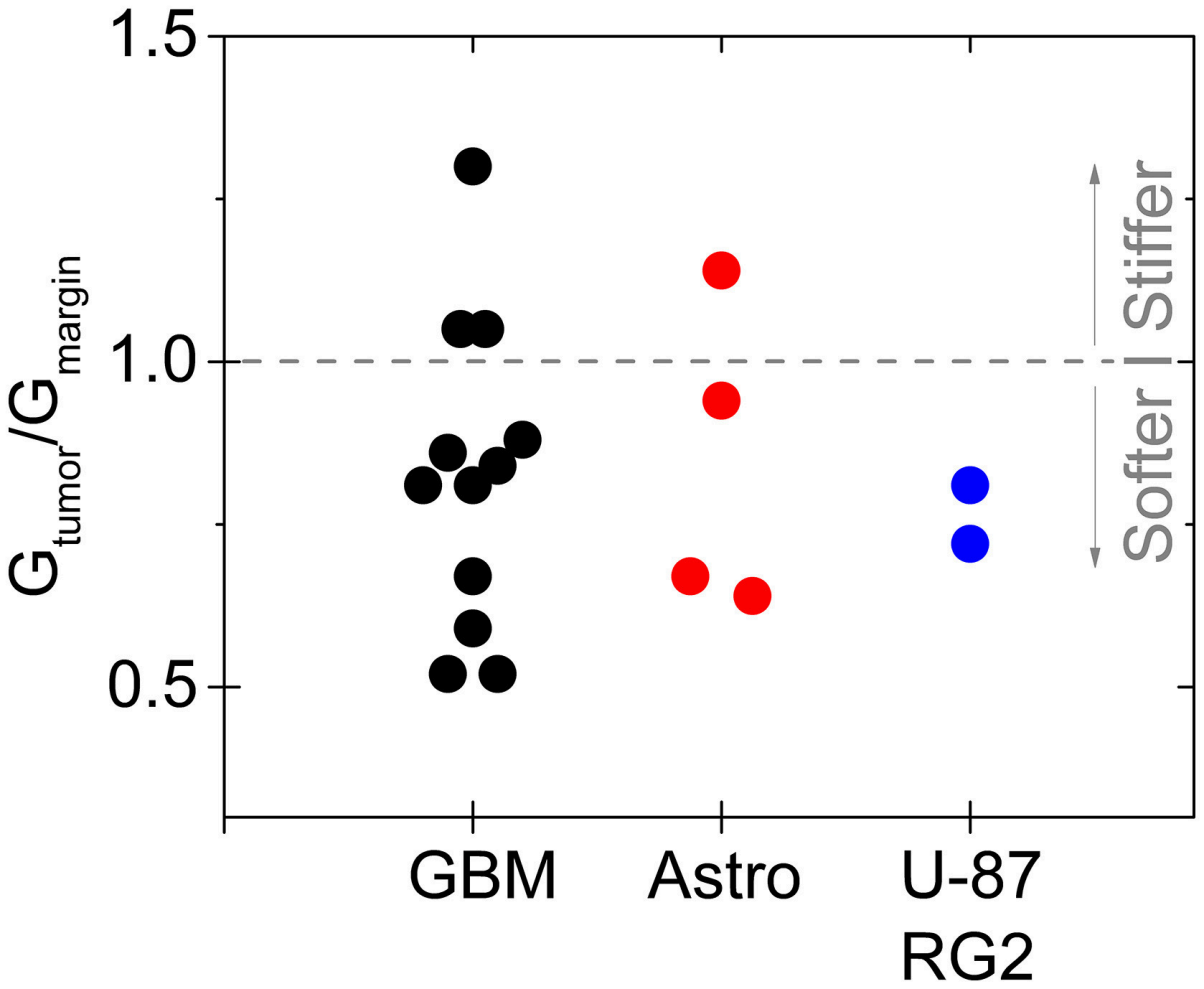
Softening of glioma and astrocytoma in humans and in a mouse model. The ratio of the stiffness of the tumor and healthy margin in human grade IV glioblastomas, grade III astrocytomas, and in the mouse tumors produced by injection of U-87 and RG2 glioblastoma cells. Based on Jamin et al. (2015), Reiss-Zimmermann et al. (2015), and Pepin et al. (2017).

In particular, gliomas do not appear to be generally stiffer than the surrounding brain tissue, either when measured *ex vivo* by indentation (Pogoda et al., 2014), or *in vivo* by MRE (Streitberger et al., 2014; Jamin et al., 2015; Chauvet et al., 2016). In one of the largest studies of gliomas by MRE, glioma stiffness showed a large variance that appeared to correlate with tumor grade (Chauvet et al., 2016), and in other studies, either of human tumors or in animal models in which different types of gliomas were produced by injection of cultured cancer cells, the tumors appear to be in many cases softer than the surrounding area (Streitberger et al., 2014; Jamin et al., 2015; Reiss-Zimmermann et al., 2015; Pepin et al., 2017; Figure 4). The structural changes leading to the softening are not yet clear, and whether *ex vivo* measurements adequately characterize the material properties *in vivo* is also not fully determined. Specifically, the properties of gliomas as well as normal brain when measured *in vitro* depends strongly on whether the sample is compressed as well as the strain magnitude at which the shear modulus is measured.

### Comparison of *in vivo* and *ex vivo* mechanical measurements

**Figure 5:**
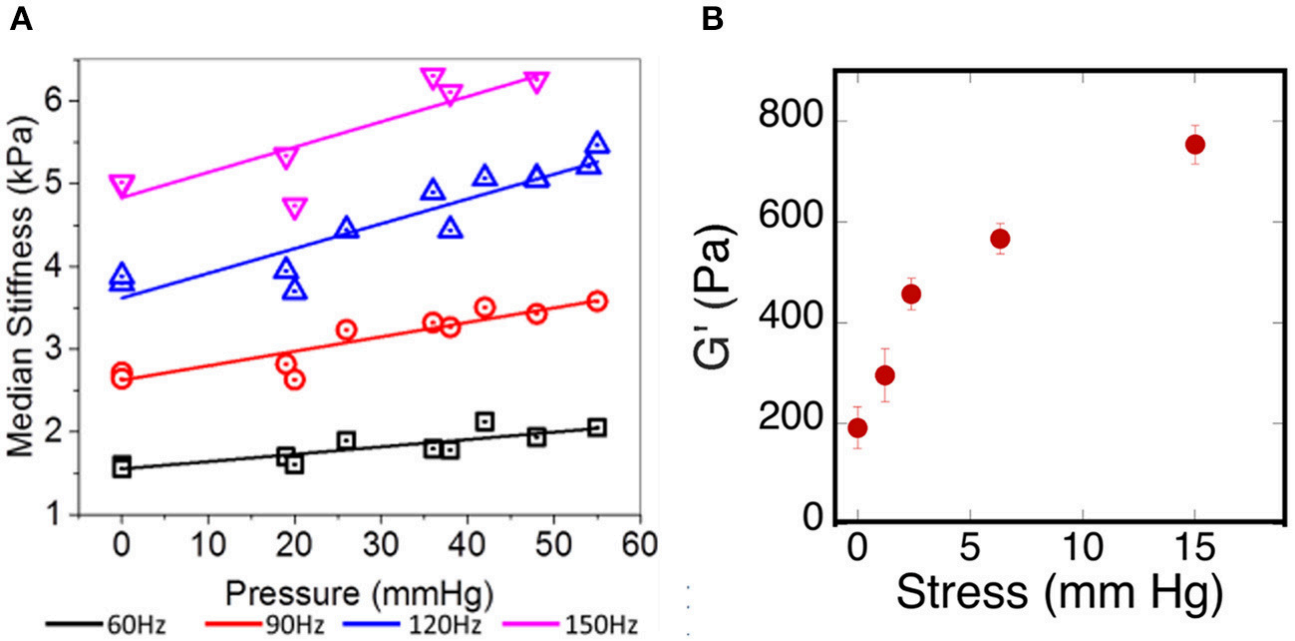
Effects of pressure on brain elastic moduli. **(A)** Adapted with permission from Arani et al. (2017), **(B)** adopted with permission from Pogoda et al. (2014).

One of the challenges in relating *ex vivo* measurements of brain stiffness to *in vivo* measurements is the fact that, once removed from the skull and after perfusion by blood and CSF ceases, the properties of the brain tissue can change if they are sensitive to the tensions and pressures that are generated *in vivo* (Xu et al., 2010; Weaver et al., 2012; Arani et al., 2017; Hetzer et al., 2017). One example of such an effect is shown in Figure 5A. Here the shear storage modulus measured by MRE is shown as a function of intracranial pressure that has been manipulated within the scull of the test animals. Increased pressure leads to an increase in elastic modulus at all of the frequencies measured. If cells within the tissue respond to local stiffness, the rigidity of the tissue they exert forces against would therefore change when intracranial pressure changes, even without any chemical alterations in the cellular or extracellular networks of the brain. This *in vivo* result is consistent with an *ex vivo* measurement showing how the shear modulus increases in brain slices subjected to uniaxial compression, as would be generated by pressure gradients of the same magnitude that were used to change stiffness *in vivo* (Figure 5B).

In addition to effects of pressure, local gradients of tension and compression also arise in the solid tissues of brain. Measurements of local internal stress made by quantifying the angle of opening after cuts are made in excised brain show that different regions of the brain are under different internal stresses (Xu et al., 2010). Specifically, white matter tracts are under tension in various regions of the brain, and these tensions need to be counterbalanced by compressive stress elsewhere, such as in the gray matter. Differences in the extent to which such stresses and stress gradients are maintained during sample preparation might be related to different conclusions from different modes of measurement as to whether white matter or gray matter is stiffer (Ichihara et al., 2001; Pervin and Chen, 2009; Christ et al., 2010; Budday et al., 2015).

**Figure 6:**
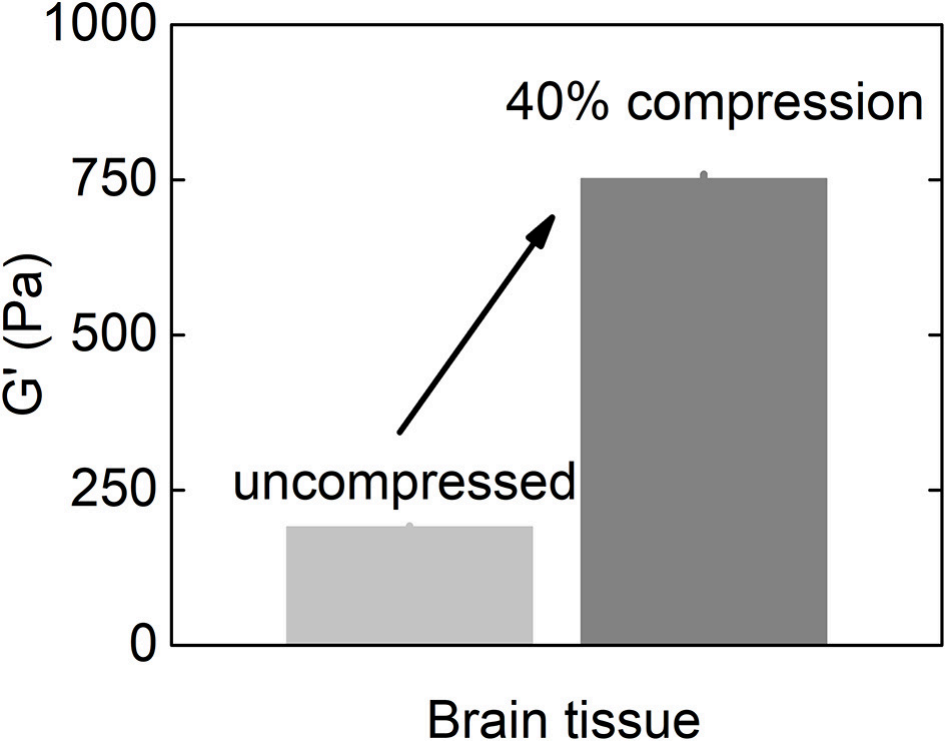
Shear storage modulus of the brain tissue at uncompressed state and when subjected to 40% uniaxial compressional stress (replotted with permission from Pogoda et al., 2014).

As described above, glioblastoma and other brain tumors are a unique category of cancers, since many reports on studies of glioma stiffness have led to different results, where both softening and stiffening of the cancerous tissue was reported. Despite discrepancy in studies of stiffness of brain-derived tumors, one important finding is the observation of increased stiffening of brain tissue in uniaxial compression presented in Figures 5, 6. The increase of storage modulus can be observed for white and gray matter, and can be as large as four times when exposed to 40% compression. Uniaxial compression of the brain tissue can mimic the effect of increased local pressure gradients within the brain that develop in glioma tumors and are reported to be in the range of 4–28 mmHg between probes placed at the wall of the tumor and ~2.5 cm distal to it (Piek et al., 1988). A uniaxial pressure gradient of this magnitude corresponds to a compressive stress of 500–3,500 Pa. These results suggest that despite the overall soft environment of the brain, cells can experience high local stiffness when glioma tumors develop and pressure gradients arise.

## ECM structure in brain

The mechano-chemical nature of the extracellular matrix plays an important role in both neurodevelopment and disease development in brain tissue (Gladson, 1999). In contrast to tumors arising elsewhere in the body, the growth of brain tumors is usually restricted only to the CNS with very low probability to metastasize to other organs (Hamilton et al., 2014). At the same time, glioblastoma cells aggressively invade the surrounding normal brain tissue, and it is postulated that the ECM of the brain stimulates glioma invasion (Bellail et al., 2004; Park et al., 2008). Although the ECM of the healthy brain is composed of many molecules that can be found in the ECM of other tissues, there are some unique properties that discriminate it. First, healthy brain possesses relatively low amounts of collagen I, the most abundant fibrous protein in vertebrates that maintains structural integrity in most other tissues. Collagen I levels can, however, be elevated in malignant gliomas (Payne and Huang, 2013). Second, brain ECM is characterized by a high content of GAGs and proteoglycans that have a large charge density and resist volume changes and assure proper hydration of tissue. Increased expression of some GAGs such as hyaluronic acid (HA) is often associated with glioma progression (Delpech et al., 1993). Therefore, the interest in fabrication of brain-mimicking ECM substrates for normal and transformed glial cells with independently defined mechanical and biochemical properties is rising.

## Mechanosensing by glial cells

**Figure 7:**
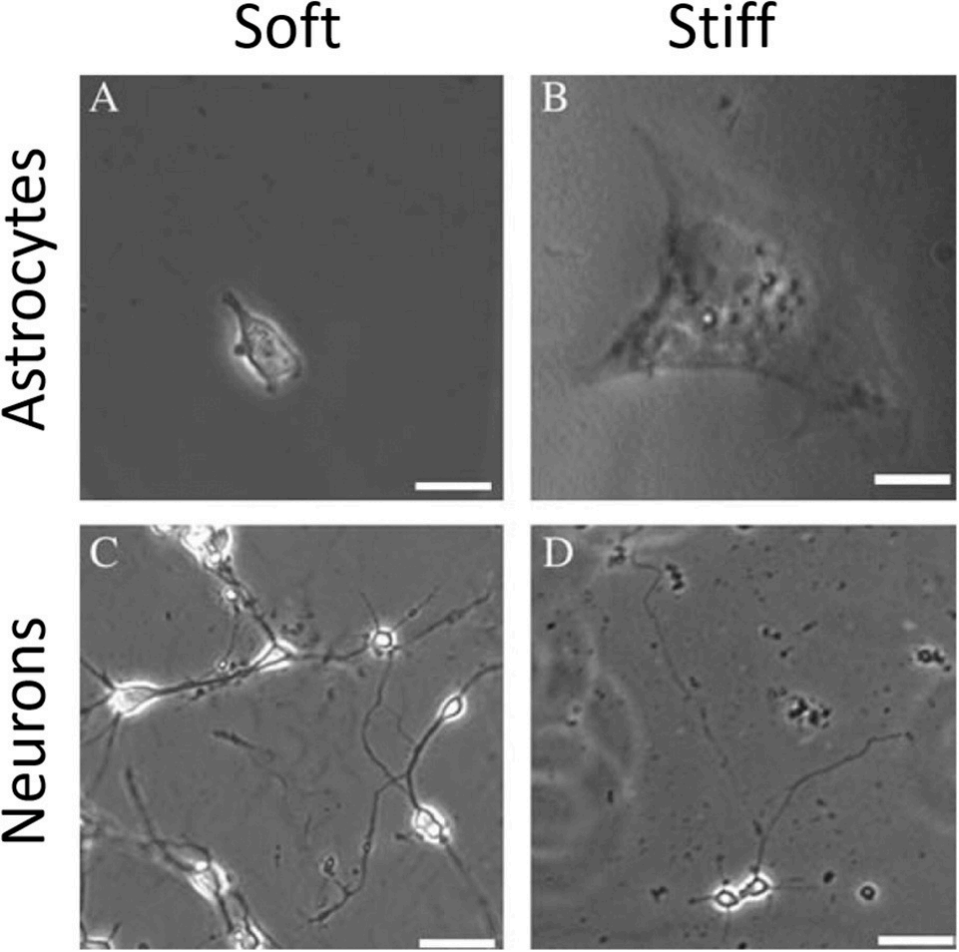
Mechanosensing of neurons and astrocytes cells. Adapted from Georges et al. (2006) (copyright 2006, with permission from Elsevier).

Several studies have shown that natural, synthetic and semi-synthetic matrices, such as polyacrylamide (PAA), fibrin, collagen I, or HA gels with varied stiffness can mimic the properties of the extracellular matrix and alter the mechanical phenotype of many cell types, including those that originate from normal brain, such as astrocytes and neurons (Georges et al., 2006; Seidlits et al., 2010; Keung et al., 2011). Similar to other cell types, astrocytes are small and round on soft gels while highly spread on stiff gels. Neurons, in contrast, show greater branching and spread morphology on soft compared to stiff substrates when grown in the presence of glia (Figure 7). A relatively sharp transition from the compliant to the rigid astrocyte phenotype was observed for substrates with shear storage moduli around 1 kPa (Moshayedi et al., 2010). The mechanical environment also influences the differentiation of rat neural stem cells (NSC), with a 500 Pa stiffness threshold causing NSCs to differentiate into neurons. Under the same conditions, studies of the proportion of neurons vs. glia in mixed cultures showed that glial cells monotonically decreased and neurons increased as the substrates became softer (Saha et al., 2008). It is worth noting, that still the classically preferred two-dimensional cell culture models, despite mimicking the stiffness of native tissues, do not reflect natural glial cells' microenvironment, thus their mechano-response for physical cues coming from the ECM and other cells of the stroma in 3D can be distinct from their response in 3D. This issue arises especially when neurodevelopment, neurodegeneration, neuroinflammation, and drug-delivery processes are being studied. Until now two types of 3D culture formats for glial cells culture have been proposed—biopolymer based hydrogels and polymer based scaffolds (Watson et al., 2017). Hydrogel 3D matrices are mostly synthesized with collagen, fibrin, or alginate and sometimes substituted with other ECM proteins like laminin and fibronectin or GAGs like HA (East et al., 2009; Andersen et al., 2015; Sreekanthreddy et al., 2015; Balasubramanian et al., 2016), and can be as soft as hundreds of Pa whereas polymer based scaffolds are often stiff and made out of polyurethane, polyamine, PDMS and other copolymer composites that can form nanofibers (Daud et al., 2012; Tiryaki et al., 2012; Puschmann et al., 2013; Smith et al., 2015). Although their usefulness in 3D culturing of glial tissue cells has been reported, standard cell analysis methods that were optimized for 2D cultures are difficult to apply.

## Mechanosensing by transformed glial cells

**Figure 8:**
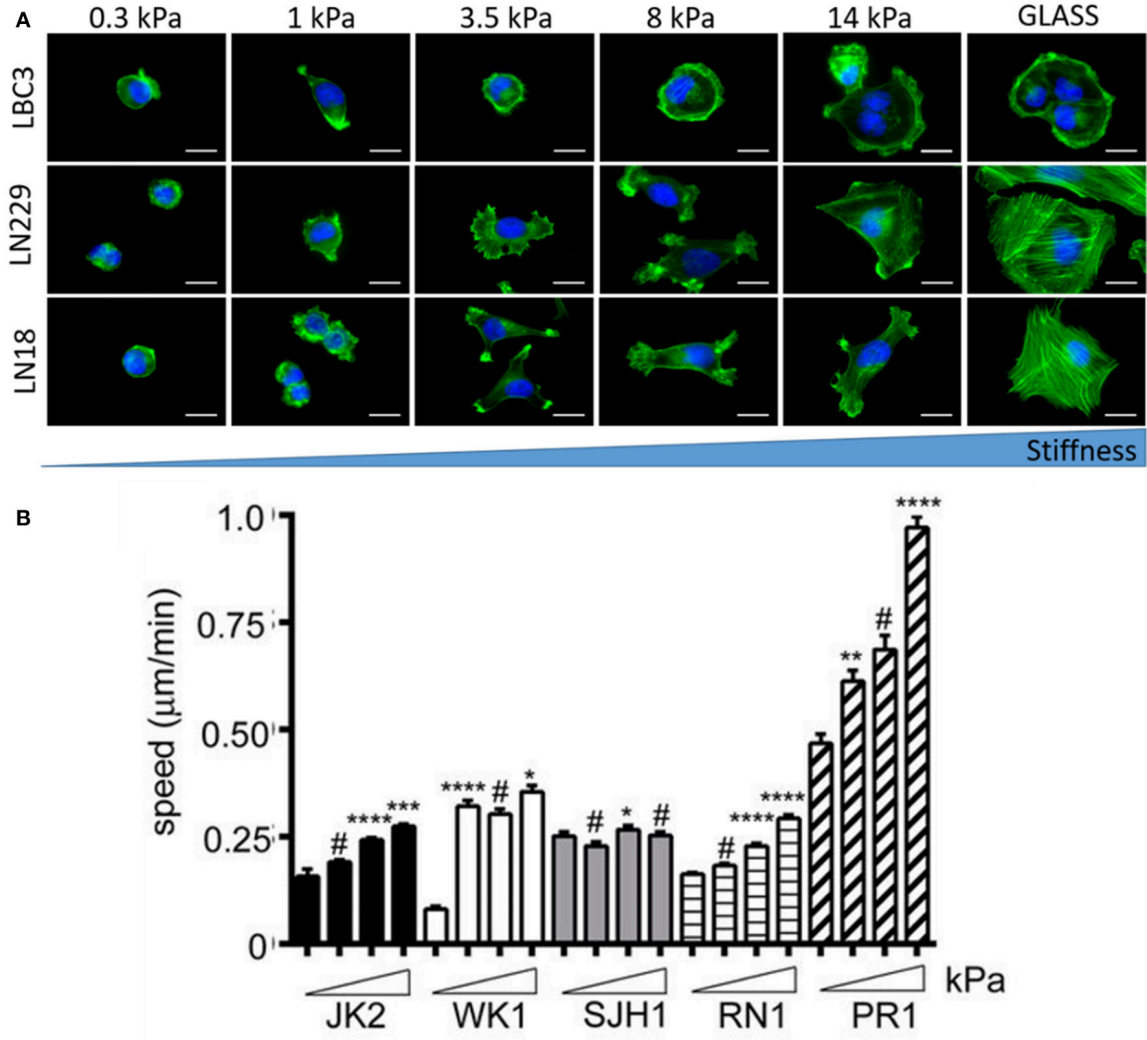
Substrate rigidity alters glioma cell morphology and motility in a cell-type specific manner. **(A)** Actin filaments (green) and nucleus (blue) staining of the LBC3, LN229, and LN18 glioblastoma cells growing on polyacrylamide substrates of different stiffness, adapted with permission from Pogoda et al. (2017) (copyright 2017, American Chemical Society), **(B)** migration speed of five different primary glioblastoma cell lines (JK2, WK1, SJH1, RN1, PR1) on polyacrylamide substrates of increasing stiffness ranging from 0.2 kPa up to 50 kPa, adapted with permission from Grundy et al. (2016). Statistical significance: *p < 0.05, **p < 0.01, ***p < 0.001, ****p < 0.0001, # not significant.

Brain malignancies such as ependymoma, medulloblastoma, and malignant glioma originate mostly form NSCs, astrocytes and oligodendrocytes but almost never from mature neurons (Liu and Zong, 2012). Differential responses of human astrocytes and transformed glioma cells to changes in substrate stiffness suggest upregulated mechanosensing in glioma cells (Pogoda et al., 2014). Mechanical heterogeneity of brain tumors is accompanied by heterogeneity in composition, since a single tumor is populated with many glioma cell subtypes that exhibit differences in morphology and proliferative potential (Soeda et al., 2015). Therefore it is important to study diverse cell populations before making general conclusions on their stiffness sensitivity. Glioblastoma cell specific response to growth on soft substrates is presented in Figure 8. Not only cellular morphology (Pogoda et al., 2017) but also migratory properties are highly specific for different cell lines with both stiffness sensitive and insensitive behavior observed (Grundy et al., 2016). Despite these discrepancies, most studies have revealed that biomechanical cues can tightly regulate glioblastoma cell behavior either by influencing cell structure, migration, proliferation, or expression and activity of contractility-mediating proteins (Ulrich et al., 2009).

There are several suggestions that uncommon aggressiveness of transformed glial cells, especially those coming from high grade astrocytomas, is regulated not only mechanically but also biochemically by brain ECM and same cellular components needed to recognize ECM cues (Bellail et al., 2004; Sulman et al., 2009). Among these components are adhesion proteins e.g., laminin, collagens, fibronectin, vitronectin, and their transmembrane integrin receptors, that allow for many essential cellular processes like adhesion, spreading, growth, migration, and gene expression (D'Abaco and Kaye, 2007), and it has been demonstrated that different adhesion proteins can alter the mechanical response of glioblastoma cells despite residing on environments with similar stiffness (Pogoda et al., 2017). In part the aggressive nature of malignant brain tumors might be attributed to the brain's unique composition with a relatively low content of fibrous proteins, balanced by very high levels of GAGs. These highly polar and negatively charged molecules tend to attract and bind water, and brain ECM consists mostly of linear chains of heparan sulfates, chondroitin sulfates, keratan sulfates, and HA (Margolis et al., 1975; Mauro et al., 1983) that can aggregate or attach to other ECM components. There are several lines of evidences that GAGs play an important role in brain malignancies, although the biochemical aspects of GAG-induced signaling is less well-understood than are signals arising from activation of integrins by protein fibers of the ECM. Efforts have been made to produce brain-mimetic ECM substrates that contains GAGs with HA-based matrices being mostly studied, due to the possibility to control their biochemical composition and mechanical rigidity (Seidlits et al., 2010; Ananthanarayanan et al., 2011; Kim and Kumar, 2014; Pogoda et al., 2017). Although the response of glioblastoma cells to substrate stiffness and composition is within the range of responses reported for other types of cells, large heterogeneity and cell-type specific reactions do not allow for straightforward definition of the role of physical cues in brain tumors development that would be uniform for all types of glioma cells.

## Conclusions

Extensive studies of rheological properties of brain tissue mechanics and brain-derived components cover the length scales and timescales from real-time *in vivo* elastography imaging, through *ex vivo* tissue rheology up to *in vitro* single cell mechanics. These studies focus on understanding the role of unique mechanical properties of brain tissue and brain cells during development, inflammation, injury, neurodegenerative diseases, and cancers. Any alteration of tissue rheology, whether it is softening, or stiffening, or altered responses of cells to these physical properties can lead to extensive pathological conditions and perturbed mechanosensing of both normal and transformed glial cells, that interact with brain ECM and reply to its mechanical cues. Despite many efforts still more rheological studies using normal and transformed glial tissues and cells are needed to relate *ex vivo* observations into *in vivo* mechanical processes.

## Author contributions

KP and PJ designed, drafted and gave final approval of the version to be submitted.

### Conflict of interest statement

The authors declare that the research was conducted in the absence of any commercial or financial relationships that could be construed as a potential conflict of interest.
